# Fixing molecular complexes in BioPAX standards to enrich interactions and detect redundancies using semantic web technologies

**DOI:** 10.1093/bioinformatics/btad257

**Published:** 2023-04-25

**Authors:** Camille Juigné, Olivier Dameron, François Moreews, Florence Gondret, Emmanuelle Becker

**Affiliations:** Univ Rennes, Inria, CNRS, IRISA—UMR 6074, F-35000 Rennes, France; PEGASE, INRAE, Institut Agro, F-35590 Saint Gilles, France; Univ Rennes, Inria, CNRS, IRISA—UMR 6074, F-35000 Rennes, France; Univ Rennes, Inria, CNRS, IRISA—UMR 6074, F-35000 Rennes, France; PEGASE, INRAE, Institut Agro, F-35590 Saint Gilles, France; PEGASE, INRAE, Institut Agro, F-35590 Saint Gilles, France; Univ Rennes, Inria, CNRS, IRISA—UMR 6074, F-35000 Rennes, France

## Abstract

**Motivation:**

Molecular complexes play a major role in the regulation of biological pathways. The Biological Pathway Exchange format (BioPAX) facilitates the integration of data sources describing interactions some of which involving complexes. The BioPAX specification explicitly prevents complexes to have any component that is another complex (unless this component is a black-box complex whose composition is unknown). However, we observed that the well-curated Reactome pathway database contains such recursive complexes of complexes. We propose reproductible and semantically rich SPARQL queries for identifying and fixing invalid complexes in BioPAX databases, and evaluate the consequences of fixing these nonconformities in the Reactome database.

**Results:**

For the *Homo sapiens* version of Reactome, we identify 5833 recursively defined complexes out of the 14 987 complexes (39%). This situation is not specific to the Human dataset, as all tested species of Reactome exhibit between 30% (*Plasmodium falciparum*) and 40% (*Sus scrofa*, *Bos taurus*, *Canis familiaris*, and *Gallus gallus*) of recursive complexes. As an additional consequence, the procedure also allows the detection of complex redundancies. Overall, this method improves the conformity and the automated analysis of the graph by repairing the topology of the complexes in the graph. This will allow to apply further reasoning methods on better consistent data.

**Availability and implementation:**

We provide a Jupyter notebook detailing the analysis https://github.com/cjuigne/non_conformities_detection_biopax.

## 1 Introduction

### 1.1 Molecular complexes and biological interactions in system biology

Understanding how biological systems adapt to their environment requires to better capture, describe, and model the interactions between their constitutive entities. With the accumulating knowledge on biological entities and their interactions, the need of a general framework to understand this information at a system level has led to a formal description of these entities and interactions. Within the context of biological pathways, several formats have been proposed such as SBML (Hucka et al. 2004, 2019) and BioPAX (Demir et al. 2010).

Biological systems typically involve an intricate network of interactions between numerous participants (Fearnley et al. 2014). Among these participants, complexes are a major class of physical entities that results from the chemical assembly of several molecules (nucleic acids, proteins, and other molecules) that bind each other at the same time and place, and form single multimolecular machines. Biologically, they play an important role in transcription, RNA splicing and polyadenylation machinery, protein export, and transport (Spirin and Mirny 2003; Zahiri et al. 2020). From the data analysis perspective, complexes cause some indirection between molecules and interactions, as molecules can participate directly to an interaction but also be a component of a complex that participates to an interaction. This introduces an additional node (the complex) between two entities that are no longer directly connected by a link in the cascade of events that triggers cell behavior.

### 1.2 Description of complexes in BioPAX

The Biological Pathway Exchange format (BioPAX;http://www.biopax.org/release/biopax-level3-documentation.pdf) is a well-established formalism to represent biological pathways at the molecular and cellular levels, including interactions (Demir et al. 2010). The BioPAX objective is to unambigously describe each component and each interaction. In the BioPAX ontology, the top four classes are Pathway, Interaction, Physical Entity, and Gene. Interactions represent the biological relationships between two or more entities, including molecular interactions, controls and conversions. Physical entities encompass small molecules, proteins, DNA, RNA, and complexes. Complexes are defined in BioPAX as “physical entities whose structure is comprised of other physical entities bound to each other noncovalently, at least one of which is a macromolecule (e.g. protein, DNA, or RNA).”

The BioPAX specification explicitly states:

complexes should not be defined recursively […] i.e. a complex should not be a component of another complex. […] Exceptions are black-box complexes (i.e. complexes in which the component property is empty), which may be used as components of other complexes because their constituent parts are unknown.

This specification about the flat representation of complexes was introduced when moving from the BioPAX1.0 to the BioPAX2.0 standard (between April and December 2005), and the rationale given for the introduction of this constraint was that the use of a tree structure could be interpreted by some users as an order in macromolecular assembly:

The reason for keeping complexes flat is to signify that there is no information stored in the way complexes are nested, such as assembly order. Otherwise, the complex assembly order may be implicitly encoded and interpreted by some users, while others created hierarchical complexes randomly, which could lead to data loss.

### 1.3 The Reactome use-case

BioPAX is based on Semantic Web technologies, with RDF facilitating integration, SPARQL facilitating querying, and OWL facilitating knowledge-based reasoning. All the major pathway databases are available in BioPAX. Among them, Reactome (https://reactome.org/) is a free, open-source, curated, and peer-reviewed pathway database (Gillespie et al. 2022). It is widely used in genome analysis, modeling, systems biology, clinical research and education, and biological pathways can be explored to shed light on interconnected proteins (Kazemzadeh et al. 2013). Despite the BioPAX specifications, we noticed the presence of recursive complexes, i.e. complexes composed of other complexes that are not black-box complexes. Importantly, these non-conform complexes were not detected by the BioPAX validator (https://biopax.baderlab.org/; Rodchenkov et al. 2013). As this pattern eludes validation, their presence in Reactome and possibly in other databases, may preclude further analyses aiming to provide robust information about mechanisms and phenotypes.

### 1.4 Motivations and results

We hypothesized that identifying and correcting recursive complexes would be valuable to enrich the biological database into interactions. This may allow a better analysis of the participants, direction, and stoichiometry of the biological interactions, either when browsing the data, or when data are processed automatically. We believe that these nonconformities in the description of the complexes are an important obstacle to the development of analysis methods that would work directly from the BioPAX format. Using SPARQL queries, we show that the well-curated Reactome database includes a large fraction of recursively defined complexes, i.e. whose components contain at least one complex (that is not a black box one). We showed that these nonconform complexes averaged one-third of the total number of complexes. Using other SPARQL queries, we corrected these recursive decompositions of complexes. Then, we showed that these corrections led to the detection of implicitly redundant complexes, whose redundancy was previously hidden by the different recursive definitions of complexes.

## 2 Approach

### 2.1 Definition of invalid recursive complexes

**Figure 1.**
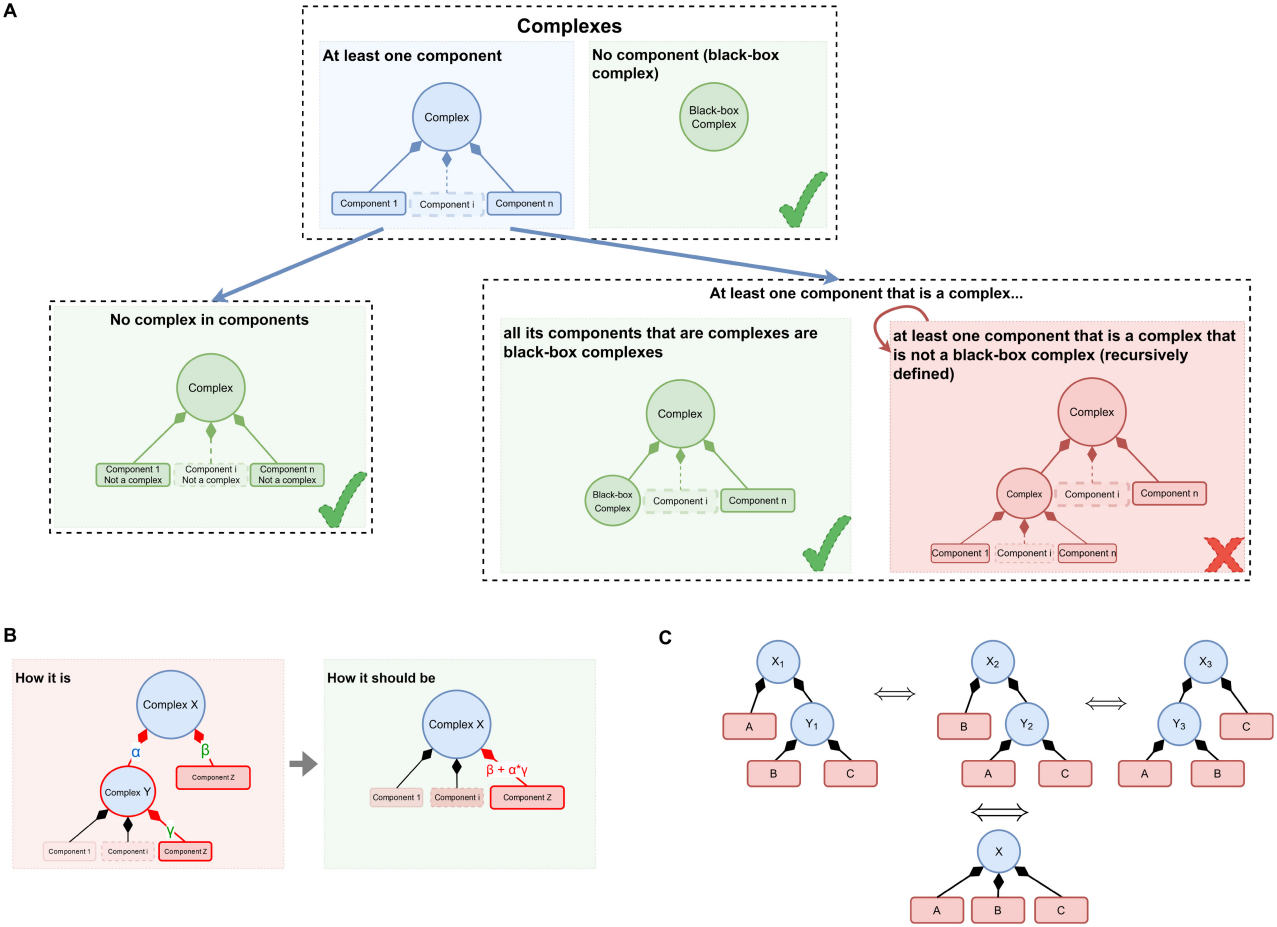
Illustration of the conformity of complexes with respect to the BioPAX specifications: identification, correction, and redundancies. (A) Validity and invalidity of the categories of BioPAX complexes. Complexes are represented by circles. Composition is represented by a diamond-head arrow from the component to its complex. A valid complex can have components that are themselves complexes only if these complexes are all black-box complexes, i.e. they do not have any components. Note that an invalid complex can itself be a component of another complex, which therefore becomes invalid as well. (B) Fixing an invalid recursive complex consists in collapsing as direct components all its direct and indirect components that are leaves in the composition tree of the complex, and then, computing the correct stoichiometric coefficient values with Equation (1). (C) Example of invalid recursive complexes leading to redundancy in the database. Fixing them made possible the detection of redundancy.

Recursive complexes are complexes whose component contains at least one complex. These recursive complexes are invalid if the inner complex is not a black-box one, i.e. the inner complex itself contains at least one component. The different categories of (in)valid complexes in BioPAX are illustrated in Fig. 1A.

### 2.2 Invalid recursive complexes in interactions

Recursive complexes can cause false negatives when identifying the interactions in which a physical entity can participate. For example, if *A*, *B*, and *C* are physical entities, *A* can directly participate in several interactions, but also indirectly when associated to *B* as a complex, or to *B* and *C* as another complex. If (*A*, *B*) and (*A*, *B*, *C*) are valid complexes, the two situations can be correctly processed by identifying the interactions matching the criterion “having a participant that is a complex composed of A.” However, if (*A*, *B*, *C*) complex is composed of the complex (*A*, *B*) and of *C*, all the interactions in which (*A*, *B*, *C*) participates would fail to meet the aforementioned criterion, because their participants are not “a complex composed of A” but “a complex composed of a complex composed of A.” We will see that in practice, such nested composition can occur over multiple levels. The approach we used to identify and fix these invalid recursive complexes, while respecting the stoichiometry where available, is illustrated in Fig. 1B.

### 2.3 Redundancies

As an additional consequence, fixing invalid recursive complexes can also result in the identification of redundancies between complexes, previously hidden by different recursive decompositions of complexes. For example, as illustrated in Fig. 1C, complexes [*A*, (*B, C*)], [(*A*, *C*), *B*], and [(*A*, *B*), *C*] could be distinct (invalid) complexes with their own identifier and at first glance, having different participants. However, they could be all fixed as a single complex having the same components *A*, *B*, and *C*.

## 3 Materials and methods

We developed semantically rich SPARQL queries for identifying and fixing invalid recursive complexes, and detecting the resulting redundancies. We applied this method on the Reactome pathways database as a use-case study [version 81 (13 June 2022)]. For the sake of reproducibility, we provide a Jupyter notebook detailing the analysis (https://github.com/cjuigne/non_conformities_detection_biopax).

### 3.1 Identifying invalid complexes

**Figure 2.**
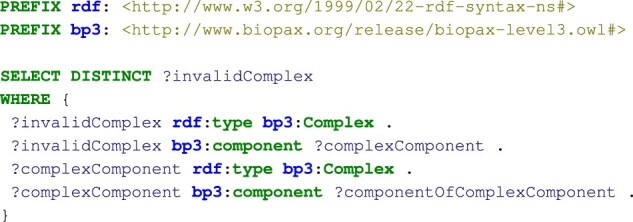
SPARQL query to identify invalid recursive complexes in BioPAX, i.e. the complexes composed of at least another complex that has components.

We first analyzed all the configurations of BioPAX complexes according to the nature of their components (1A). The SPARQL query presented in Fig. 2 allows to identify the invalid recursive complexes. Other SPARQL queries to identify non-recursive complexes and valid recursive complexes, are presented in the Jupyter notebook.

### 3.2 Fixing the invalid complexes

Fixing an invalid recursive complex can be decomposed with a four steps methodology: (i) collapsing as direct components all its direct or indirect atomic components (i.e. those that are not complexes or black-box complexes); they correspond to the leaves in the tree of components, (ii) deleting all the other components, (iii) setting the correct values for the stoichiometric coefficients, and (iv) preserving all other attributes of the complex. The whole procedure consisting of python scripts and SPARQL queries is available in the Jupyter notebook.

To collapse the direct components of the complex in steps (i) and (ii), the original BioPAX relation component was replaced by a component relation between the root complex and its leaves in the tree of components. We kept all the other relations of the complex in step (iv).

To compute the stoichiometric coefficients in step (iii), we traced the stoichiometric coefficients from each leaf up to the root complex. We also considered the fact that a physical entity can be a component of several parts of the recursive complex. Tracing stoichiometry is illustrated in Fig. 1B where complex *Y* was composed of *γ Z*, and *X* was itself composed of *α Y*. This resulted in α×γ*Z* in *X* (*via Y*). If, in addition to being a component of *Y*, *Z* was also a direct component of *X* with *β* as stoichiometric coefficient value, this resulted in α×γ+β occurrences of *Z* in *X*.

We noted *S_y_*(*z*) the global stoichiometric coefficient value of *Z* at *Y*, i.e. the number of occurrences of *Z* in *Y*, and *C*(*y*) the set of the direct components of *Y*. Formula 1 recursively computes the stoichiometric coefficient value of any physical entity *Z*.


(1) $$\{\begin{array}{cc} S_{z}\left(z\right)=1 & \\ S_{y}\left(z\right)=0 & if\;\left(y\neq z\right)\wedge \left(C\left(y\right)=\emptyset \right)\\ S_{y}\left(z\right)=\sum_{p\;\in \;C\left(y\right)}S_{y}\left(p\right)\times S_{p}\left(z\right) & \text{otherwise} \end{array}$$

### 3.3 Identifying redundant complexes

We considered as redundant the complexes that have exactly the same components with the same stoichiometric values and the same cellular location. Figure 1C illustrates how invalid recursive complexes can be the cause of redundancy due to the order by which the components are nested in the complexes. Fixing the invalid complexes made possible the detection of redundancy. For that, we developed a SPARQL query that identifies the pairs of complexes that have the same components and properties but different identifiers. This query is also available in the Jupyter notebook.

## 4 Results

### 4.1 Invalid complexes represent a significant part of complexes in the BioPAX description of Reactome

The Human subset of Reactome v81 is composed of a total of 14 987 complexes. Among them, we identified 862 black-box complexes (i.e. complexes without any components). Among the remaining 14 125 complexes with at least one component, 8292 complexes have no component that is itself a complex with components. Together with the 862 black-box complexes, they represent the 9154 valid complexes.

On the opposite, we identified 5833 complexes that have at least one component that is a complex with components. Altogether, these 5833 invalid recursive complexes represent 39% of the 14 987 complexes in Reactome. None of them have been detected by the BioPAX-validator tool (Rodchenkov et al. 2013).

These invalid recursive complex participate to 7149 out of the 22 237 interactions identified in Reactome (i.e. 32%).

### 4.2 Fixing invalid complexes increases the average number of components participating to complexes in Reactome

All the 5833 invalid complexes were fixed thanks to a python script and SPARQL queries (3.2). After fixing recursive complex description, all components of the complexes are described by a flat representation in accordance with the BioPAX specification.

**Figure 3.**
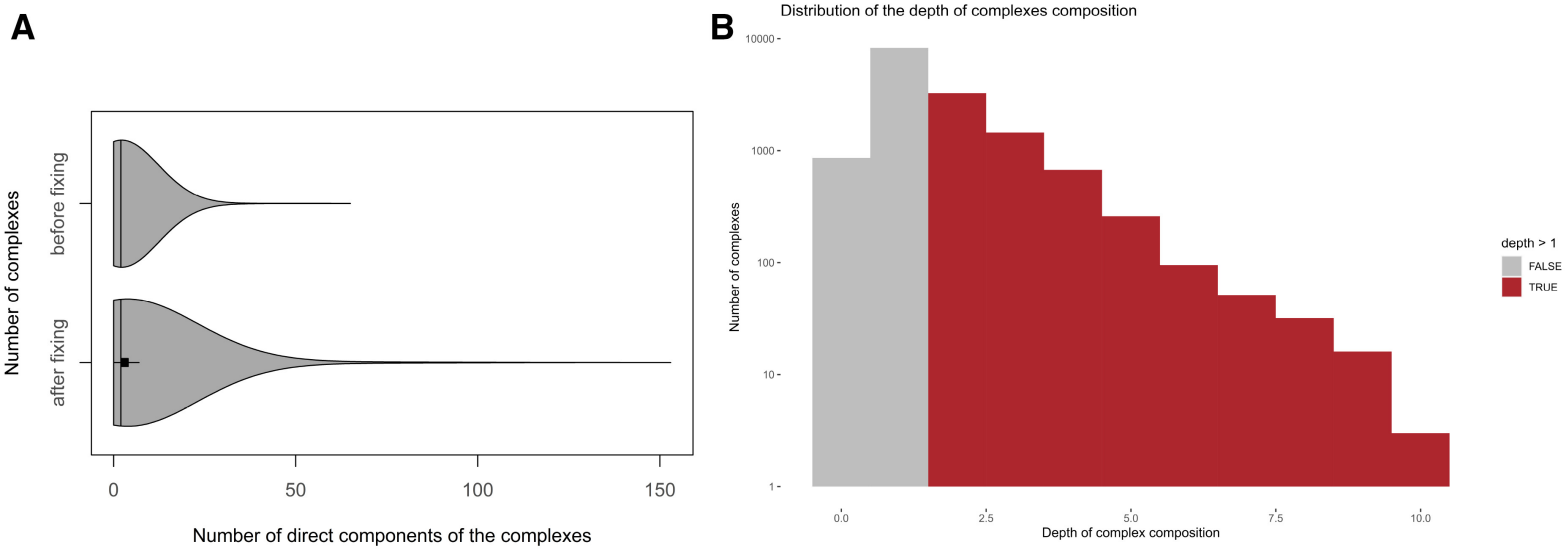
Comparison of topological features before and after fixing invalid complexes. (A) Number of direct components of the complexes in the Human dataset of Reactome v79 before (top) and after (bottom) fixing invalid recursive complexes. Fixing invalid complexes clearly increased the number of components counted in many complexes. (B) Distribution of the depth of recursive complex definitions in the initial Human dataset of Reactome v81 (logarithmic scale).

As expected, with this flat representation, the number of direct components implicated in a complex increases (paired *t*-test, *P *<* *.0001). Indeed, in the initial Reactome dataset, the average number of direct components in a complex is 2.2 (σ=2.6) and the complexes with the largest number of components are R-HSA-5626171 and R-HSA-72069, each having a maximum of 65 components. After fixing the invalid complexes, the average number of direct components in a complex is 4.3 (σ=8.7) and the largest complex is R-HSA-156656 with 151 components. The most drastic changes concern complexes R-HSA-927767 and R-HSA-927890 which both move from 3 to 103 direct components. Figure 3 illustrates the number of direct components identified before and after fixing the invalid complexes. The distribution of the gain in the number of direct components is available in Supplementary Fig. S1.

### 4.3 Fixing invalid complexes reduces the path length from a complex to each of its components

We studied the composition depth of the complexes, before and after fixing the invalid ones. For a given complex, its composition depth was measured as the maximal length path between the root complex to its leaves. As illustrated in Fig. 3A, the tree-like definition of invalid complexes artificially increases the depth of the complex composition.

In Fig. 3B, the depth of complex composition is represented before the fixing procedure. Valid complexes have a depth of either one, or zero when they are black-box complexes. The red part of the figure represents invalid complexes having a depth greater than one. The maximum depth is 10, which leads to an artificial extension of the path length from the root complex to the majority of its leaves (example of R-HSA-68 466 is given in Supplementary Fig. S2).

As expected, the correction of invalid complexes repairs the topology by reducing the path length between a complex and its components to a maximal length of 1. The depth of all complexes is 0 (black-box complexes) or 1 (complexes with components).

### 4.4 Fixing invalid complexes improves the detection of redundant complexes

Redundancies are detected between entity pairs but can also occur between more than two entities, as illustrated in Fig. 1C with three equivalent complexes. In this example, three pairs of redundancies (*X*_1_, *X*_2_), (*X*_2_, *X*_3_), and (*X*_1_, *X*_3_) are thus detected, corresponding to the size of a maximal clique with three vertices.

Among the 14 987 complexes from the original Reactome database, the SPARQL query identifies 137 pairs of redundant complexes involving 249 distinct complexes. They constitute 121 maximal cliques of equivalent complexes (clique size ranging from 2 to 6 complexes), corresponding to 128 complexes in excess. In other words, we highlighted 121 groups of 2 to 6 redundant complexes identified by searching for pairs of redundant complexes. These redundancies are explicit, as they are not masked by a tree-like definition of complex components.

**Figure 4.**
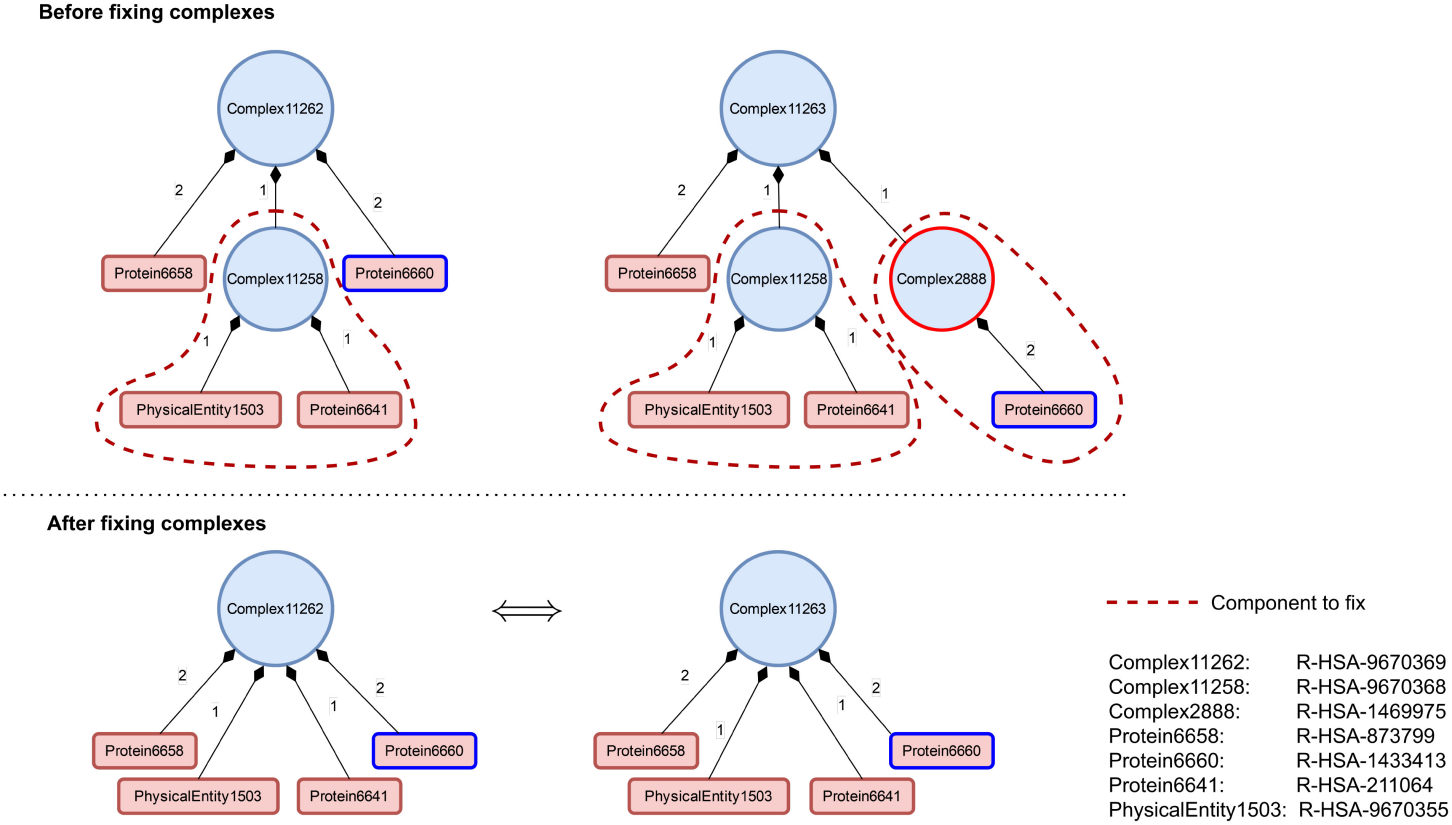
Original invalid compositions of Complex11262 (R-HSA-9670369) (top left) and Complex11263 (R-HSA-9670372) (top right) in Reactome. Some components are complexes that should be replaced by a flat definition of components (indicated with dashed red lines). The fixed versions (bottom left and right, respectively) have a greater number of direct components than the original. Both fixed versions have the same components with the same stoichiometric coefficients, which reveal their redundancy. The structure difference between the original versions is highlighted in red: Complex2888 (R-HSA-9670372) is composed of an intermediate dimer of the p-STATs protein Protein6660 whereas Complex11262 (R-HSA-9670369) is directly composed of p-STATs Protein6660 with a stoichiometric coefficient of 2. A more detailed figure is available in Supplementary Fig. S3.

After fixing the invalid complexes, we identified 217 pairs of redundant complexes involving 347 distinct complexes. They constitute 164 maximal cliques of equivalent complexes (clique size ranging from 2 to 6), corresponding to 183 complexes in excess. The fixing procedure, by replacing the tree-like definition of complexes by a flat description of complexes, thus allows to detect more redundancies. These redundancies are implicit, as they are masked by a tree-like definition of complex components. They become explicit with the flat description of complex components. Figure 4 illustrates how fixing structurally different complexes reveals this redundancy.

Reactome contains cross-references to ComplexPortal (Meldal et al. 2021) to annotate complexes. We sought to verify the possibility of identifying complex redundancies in Reactome without our SPARQL query-based procedure, by simply and unambiguously identifying complexes via their identifiers in a specialized database dedicated to complexes such as ComplexPortal, even if ComplexPortal incompletely supports stoichiometry, which may be a severe limitation. Because of the very modest size of the ComplexPortal Database (1429 complexes for *Homo sapiens*), ComplexPortal IDs do not allow to detect any redundancies identified with the SPARQL query: among the 347 complexes that constitute 217 pairs of redundant complexes identified, only 3 out of 347 have a ComplexPortal ID.

This study is available in a Jupyter notebook on the GitHub repository. Supplementary Table S1 lists all symetric hasSameCompositionAs relations between cliques of redundant complexes. The corresponding.ttl files are available on the GitHub repository (https://github.com/cjuigne/non_conformities_detection_biopax).

### 4.5 Application to nonhuman organisms in the Reactome database

The complete procedure was then applied to all other organisms available in Reactome, to determine whether the large fraction of nonconform complexes detected in 4.1 (39%) are specific or not to the well-explored Human dataset. The results are presented in Table 1. This shows that the large fraction of invalid complexes is not restricted to the Human dataset but also accounts for all of the 13 tested species. Datasets from all species exhibit between 30% (*Plasmodium falciparum*) and 40% (*Sus scrofa*, *Bos taurus*, *Canis familiaris*, *Gallus gallus*) of recursively defined invalid complexes. For all these species, repairing the topology of the complexes in the same way as for *H.sapiens* significantly increases the average number of direct components of the complexes. With the flat representation of complexes, we also observed complex redundancies in complexes for each organism, although not in the same proportion as in the Human dataset (from 2 cliques for *P.falciparum* to 16 cliques for *Mus musculus*).

**Table 1.** For each organism in the Reactome database, we counted the total number of complexes, then we identified the number of invalid complexes and evaluated the average number of direct components and the number of redundant complexes (pairs and cliques), before and after fixing the invalid complexes.

Organism	Total number of complexes	Invalid complexes, *n* (%)	Average number of direct components		Redundancy	
			Before fixing	After fixing	Before fixing	After fixing
*Homo sapiens*	14 987	5833 (39)	2.2 (SD 2.6) max 65	4.3 (SD 8.7) max 151	137 (121 cliques)	217 (164 cliques)
*Mus musculus*	10 707	4235 (39)	2.3 (SD 2.9) max 65	4.5 (SD 9.0) max 151	2 (2 cliques)	16 (16 cliques)
*Sus scrofa*	9022	3638 (40)	2.3 (SD 2.9) max 65	4,7 (SD 9,4) max 151	0 (0 clique)	12 (12 cliques)
*Bos taurus*	9412	3773 (40)	2.3 (SD 2.9) max 65	4.6 (SD 9.2) max 147	0 (0 clique)	12 (12 cliques)
*Saccharomyces cerevisiae*	1662	517 (31)	2.5 (SD 3.3) max 50	6.0 (SD 12.4) max 106	1 (1 clique)	5 (5 cliques)
*Caernorhabditis elegans*	4350	1560 (36)	2.4 (SD 3.3) max 64	5.0 (SD 10.4) max 149	0 (0 clique)	8 (8 cliques)
*Canis familiaris*	8945	3601 (40)	2.3 (SD 3.0) max 65	4.7 (SD 9.4) max 151	0 (0 clique)	12 (12 cliques)
*Danio rerio*	8618	3391 (39)	2.3 (SD 3.0) max 65	4.7 (SD 9.5) max 150	0 (0 clique)	11 (11 cliques)
*Dictyostelium discoideum*	2366	792 (33)	2.4 (SD 2.8) max 50	5.3 (SD 9.8) max 103	0 (0 clique)	3 (3 cliques)
*Drosophilia melanogaster*	5361	1955 (36)	2.4 (SD 3.0) max 64	4.8 (SD 9.7) max 149	2 (2 cliques)	9 (9 cliques)
*Gallus gallus*	8046	3244 (40)	2.3 (SD 2.8) max 65	4.7 (SD 9.4) max 149	2 (2 cliques)	13 (13 cliques)
*Plasmodium falciparum*	875	264 (30)	2.4 (SD 3.6) max 50	5.4 (SD 11.9) max 103	0 (0 clique)	2 (2 cliques)
*Rattus norvegicus*	9645	3780 (39)	2.3 (SD 2.9) max 65	4.5 (SD 9.2) max 151	4 (4 cliques)	15 (15 cliques)

## 5 Discussion and perspectives

In this study, we show that nonconform recursive complexes affect a large proportion of Reactome database exported in the BioPAX format. Indeed, they constitute 30%–40% of the complexes for all organisms, and participate to about one-third of the interactions. The fact that this phenomenon occurs in all Reactome organisms may be explained in part by the fact that some interactions and complexes in nonhuman species are inferred if a large fraction (at least 75%) of the proteins involved in the interactions or complexes have an ortholog for the species considered in PANTHER. Thus, taking into account corrections for *H.sapiens* prior to PANTHER inference process will probably result in a diminution of the phenomenon for other species. Due to this pervasiveness, any solution to overcome or repair recursive complexes would be valuable for automated graph analysis whatever the biological questions to be addressed.

In addition to being widely present in the datasets, we also show that invalid complexes composition reaches up to 10 levels in the tree of components of the complex. In these situations, navigating in the BioPAX file from some components of the complete complex to the complete complex is both painstaking and detrimental to computational performance.

Fixing recursive complexes consists in adding all the indirect components of a complex as new direct components. This leads to two main outcomes. First, it helps to reduce the path length from a complex to its components, since the maximal path length with the conform topology is now 1 (which solves the navigation limitation identified previously). Second, this increases the average number of components participating to complexes in Reactome. In the human dataset, this correction doubled the average number of direct components of a complex, from 2.2 to 4.3 components. This result is not specific to the human dataset: for all other organisms, the number of direct components has doubled.

As a side effect, the procedure reveals redundancies between complexes. These redundancies would have been would have been difficult to identify without the procedure, because they are masked by the recursive definition of complexes. This is particularly relevant for the Human dataset, since the redundant complexes increase from 137 to 217 redundant pairs (+58%) before and after the fixing procedure. For the identification of redundant complexes, the verification that the cellular location is the same is crucial. Indeed, without considering cellular location, 243 pairs of redundant complexes can be identified whereas taking into account cellular location reduces this number to 217 pairs of redundant complexes. This difference is caused by the fact that physical entities can exist several times in BioPAX as long as they refer to physical entities in different states (including modifications, cellular location, etc.).

The BioPAX ontology, as defined in Demir et al. (2010), is a very powerful format to represent and unify all the subtile levels of interactions occurring in biological pathways. It exploits the ontology formalism to conciliate genericity (with the high-level entity classes Physical Entity and Interactions) and a precise description of the processes (with low-level classes and various properties). However, this is also a quite complicated format description, and induces a computational complexity when reasoning on the data structured in this format. Strömbäck and Lambrix (2005) anticipated this, stating that:

This makes it possible to benefit from reasoning and conclusions based on the semantics given by OWL and the ontology, but the cost is a higher computational complexity for reasoning and integration of data.

We have showed that more recent computational advances such as SPARQL, which is designed to handle ontologies, are better adapted to this task and can be also scaled up.

Taking advantage of SPARQL queries, the procedures developed herein to (i) fix recursively defined complexes and (ii) identify redundant complexes, allow to correct the noncompliance with BioPAX specifications. As these procedures are applied at the level of the BioPAX files, they can be applied to all other major BioPAX pathway databases, including KEGG (Kanehisa and Goto 2000), MetaCYC (Caspi et al. 2020), PathwayCommons (Cerami et al. 2010), and WikiPathways (Martens et al. 2021), to assess the importance of invalid recursive complexes in these databases. Our strategy to modify directly the BioPAX files also ensures that further analyses of biological networks can be processed without any need to modify any standard queries or scripts based on BioPAX libraries such as Paxtools (Demir et al. 2013) or PyBioPAX (Gyori and Hoyt 2022).

## Supplementary Material

btad257_Supplementary_Data
