# Supplementary material for: Fixing molecular complexes in BioPAX standards to enrich interactions and detect redundancies using semantic web technologies
Source: Bioinformatics. 2023 Apr 25;39(5):btad257. doi: 10.1093/bioinformatics/btad257 (PMC10168583; doi:10.1093/bioinformatics/btad257)
Supplement: btad257_Supplementary_Data [file btad257_supplementary_data.pdf]

Fixing molecular complexes in BioPAX standards to enrich interactions and detect redundancies using Semantic Web technologies

Camille Juigné, *et al.*

SUPPLEMENTARY DATA (TABLES AND FIGURES)

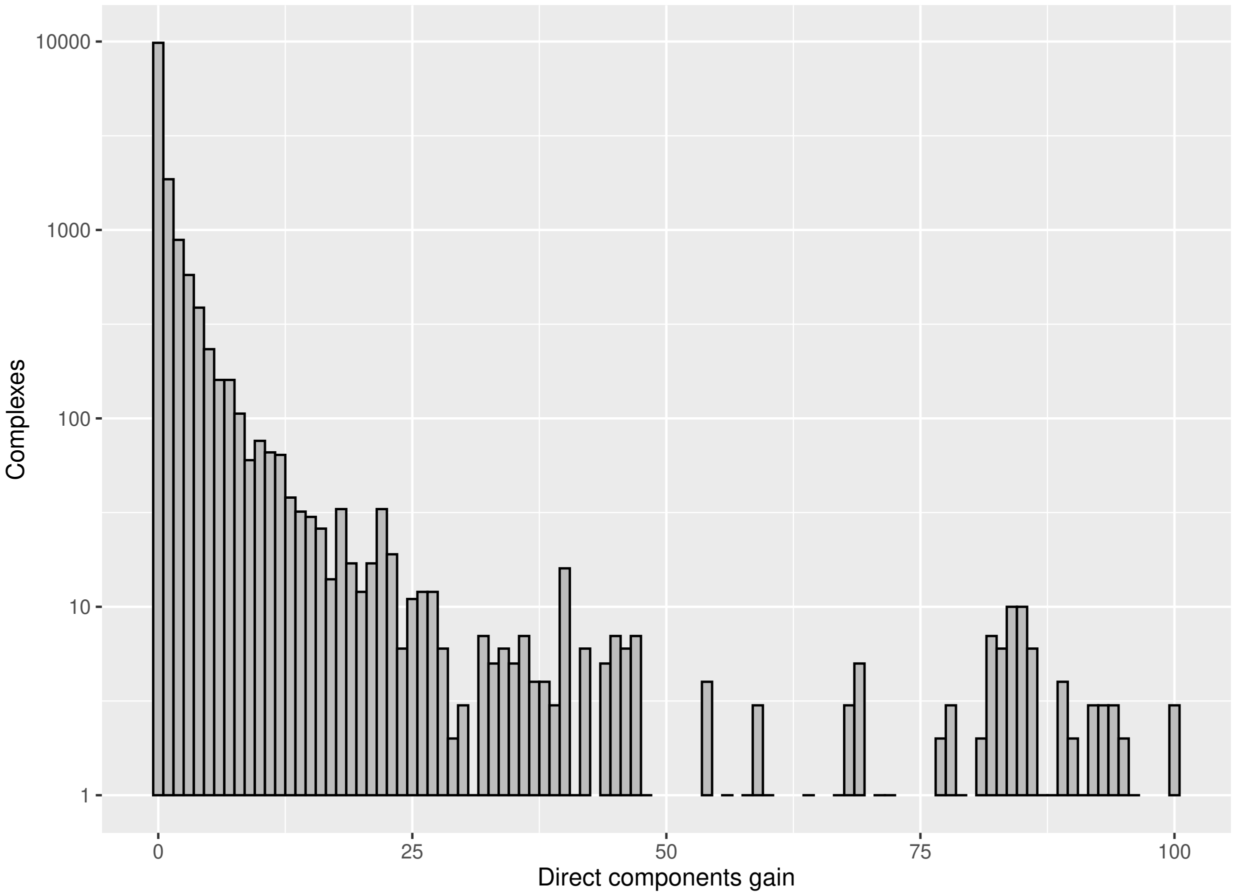

Figure S1: Distribution of the gain in the number of direct components.

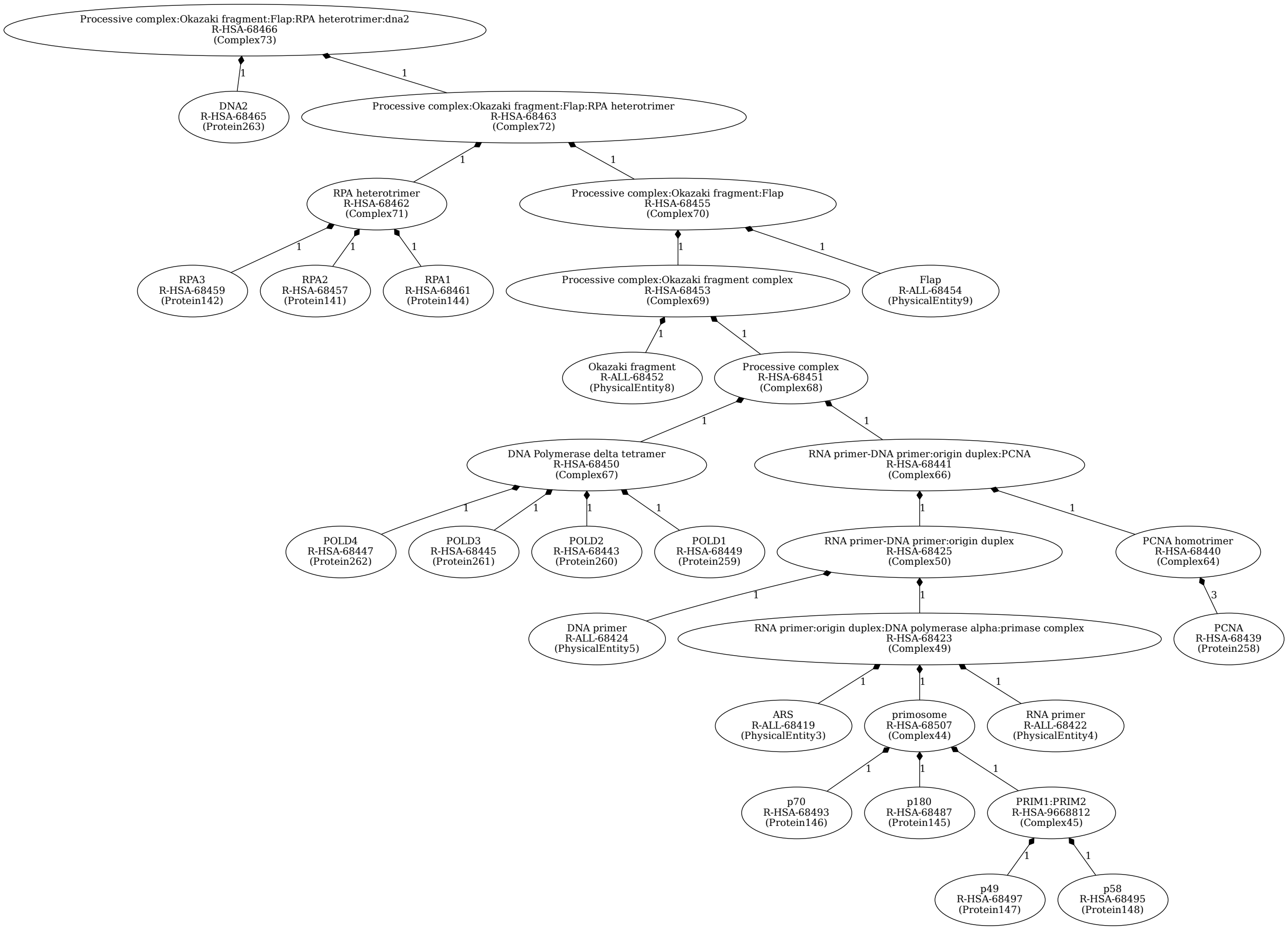

Figure S2: Representation of the nested complex R-HSA-68466 of depth 10.

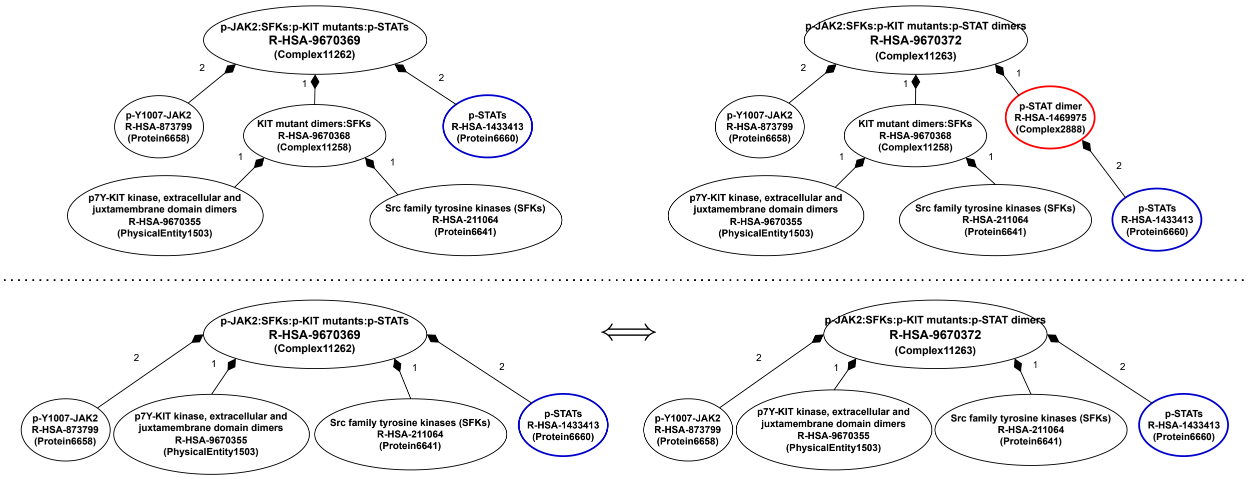

Figure S3: Original invalid compositions of Complex11262 (R-HSA-9670369) (top left) and Complex11263 (R-HSA-9670372) (top right) in Reactome. The fixed versions (bottom left and right, respectively) have a greater number of direct components than the original. Both fixed versions have the same components with the same stoichiometric coefficients, which reveals their redundancy. The structure difference between the original versions is highlighted in red: Complex2888 (R-HSA-9670372) is composed of an intermediate dimer of the p-STATs protein Protein6660 whereas Complex11262 (R-HSA-9670369) is directly composed of p-STATs Protein6660 with a stoichiometric coefficient of 2.
